# Catalytic Ozonation of Reactive Black 5 in Aqueous Solution Using Iron-Loaded Dead Leaf Ash for Wastewater Remediation

**DOI:** 10.3390/molecules29040836

**Published:** 2024-02-13

**Authors:** Latif Hussain, Farhan Javed, Muhammad Wasim Tahir, Hafiz Muhammad Shahzad Munir, Amir Ikhlaq, Anna Wołowicz

**Affiliations:** 1 Department of Chemical Engineering, University of Engineering and Technology, Lahore 54890, Pakistan; latifshifai@gmail.com (L.H.); farhan.javed@uet.edu.pk (F.J.); wasim.tahir@uet.edu.pk (M.W.T.); 2 Department of Chemical Engineering, Khawaja Fareed University of Engineering and Information Technology, Rahim Yar Khan 64200, Pakistan; 3 Institute of Environmental Engineering and Research, University of Engineering and Technology, Lahore 54890, Pakistan; aamirikhlaq@uet.edu.pk; 4 Department of Inorganic Chemistry, Faculty of Chemistry, Institute of Chemical Sciences, Maria Curie Sklodowska University, Maria Curie-Sklodowska Square 2, 20-031 Lublin, Poland

**Keywords:** catalytic ozonation, dead leaves ash, Reactive Black 5, wastewater

## Abstract

In the current study, iron-loaded dead leaf ash (Fe-DLA) was used as a novel catalyst in the heterogeneous catalytic ozonation process (HCOP) for textile wastewater containing Reactive Black 5 (RB-5). The research demonstrates a significant boost in removal efficiency, reaching 98.76% with 1.0 g/min O_3_ and 0.5 g/L catalyst dose, by investigating key variables such as pH, ozone and catalyst doses, initial concentration, and the presence of scavengers in 1 L wastewater. The addition of tert-butyl alcohol (TBA) reduced RB-5 elimination, indicating the involvement of OH radicals. Catalyst reusability decreased slightly (2.05% in the second run; 4.35% in the third), which was attributed to iron leaching. A comparison of single ozonation (Fe-DLA) adsorption and catalytic ozonation processes (Fe-DLA/O_3_) revealed that the combined process improved dye degradation by 25%, with removal rates ranking as Fe-DLA adsorption O_3_ Fe-DLA/O_3_, with an impressive 76.44% COD removal. These results strongly support RB-5 removal using Fe-DLA and HCOP at a basic pH, highlighting the catalyst’s utility in practical wastewater treatment.

## 1. Introduction

The rapid and expansive surge in industrialization over the past few decades has triggered a substantial increase in the demand for freshwater resources. The discharge of a considerable volume of non-biodegradable and highly toxic organic pollutants into various aquatic ecosystems causes a significant deterioration in water quality and reduces the amount of water available for drinking. These pollutants encompass a broad spectrum of harmful substances, including pharmaceutical compounds [1,2], different types of dyes used in industrial processes [3,4], and potent pesticides employed in agriculture and pest control activities [5]. This multifaceted issue underscores the critical need to address the intricate interplay between industrial expansion, water consumption, and the pervasive release of hazardous substances that pose significant ecological and public health concerns [6,7,8]. The textile industry is a major global industrial sector, which uses a huge amount of water during dyeing and finishing. Wastewater from the textile industry contributes almost 80% of the overall industrial wastewater discharge and includes different types of dyes that are highly hazardous to the environment (annual dyes production: >100,000 types; weight > 700,000 tons) [7,8,9]. Two-hundred-and-eighty-thousand tons of the global production of dyes directly enters wastewater [8]. Reactive dyes are the most applied due to their brightness, ease of application, fastness, and the fact that this type of dye is recommended for cellulosic materials, which still form the largest percentage of materials used in the textile industry [9]. Reactive dyes can efficiently covalently bond with the hydroxyl groups in the cellulose, resulting in the best and fastest color [10]. Reactive Black 5 is one such dye, which is extensively applied in the textile industries [11]. These organic contaminants contribute to odor and color and enhance the chemical oxygen demand (COD) in the water effluent [7].

During the dyeing process, some of the dyes are hydrolyzed before reaction with the cellulose and are physically attached to the fiber, either by adsorption or by absorption. Such dyes need extensive cleaning during the wash-off step, or else they may leach even during the product’s usage, putting the end-user’s health at risk. After the dyeing process, the fabrics are extensively rinsed in the wash-off process, which is considered to be the most water-consuming step in the textile manufacturing industry [12]. The wash-off process is completed in many cyclic steps to ensure that the fabric is thoroughly cleaned [10]. The resultant wastewater produced in this step contains residual dyes, additives, and other chemicals, which are hazardous to aquatic life and other living beings. Such contaminants, if discharged without proper treatment, may lead to the severe deterioration of our ecosystem, affecting human lives [13]. Stringent regulations have been established, along with restrictive environmental policies, to cope with the scarcity of fresh water, leading to the introduction of sustainable manufacturing methods in several industries, including the textile sector. Many conventional methods have been adopted to conserve water and reduce wastewater generation, including electrocoagulation, adsorption, and ozonation [14,15] (Figure S1). There are various advantages of using ozone as an oxidant, including its high oxidation potential and ease of dosing as a gas [16]. The treatment capacity is enhanced when the ozonation process is aided by a heterogeneous catalyst. In recent years, some advanced oxidation processes (AOPs) were developed that appear promising for the removal of recalcitrant contaminants [7,17,18,19].

The heterogeneous catalytic ozonation process is one of the treatment processes that has been effectively applied for the removal of different contaminants from industrial wastewater, among other dyes, and this process has shown great potential for further development. Ozone is a strong oxidant, but it is preferred not to use it alone due to its selectivity and the formation of intermediates. Adding a catalyst to the ozone treatment leads to the formation of hydroxyl radicals, which are more efficient [20,21,22,23]. The selection of a suitable catalyst plays a major role in the optimization of the process because it affects the ozone’s decomposition and the formation of hydroxyl radicals [24,25,26,27]. Pakistan, being an agriculture-rich country, produces a huge amount of waste biomass annually, which is mostly burnt and discarded. Alternatively, it can be converted from waste to another resource, providing a sustainable solution. The abundant availability of *Sapindus mukorossi* trees in the local area and the regular collection of dead waste leaves make them a key potential resource [28].

To the best of the authors’ knowledge and as the literature data indicate (Table S1) [29,30,31,32], the present study is the first application of the iron-loaded *Sapindus mukorossi* dead leaf ash catalyst in Reactive Black 5 treatment in the heterogeneous catalytic ozonation process. In previous studies involving the catalytic ozonation process, extensive research was conducted utilizing various inorganic materials as catalysts, such as activated carbon [33], clays, zeolites [34], metal oxides [35], and graphene oxides [36]. However, these materials are expensive and have some limitations in their application. Therefore, it is indeed important to investigate the application of novel materials as catalysts in such processes. Waste-biomass-based materials offer an economic and environmentally friendly solution with abundant availability. There has been limited research reporting on the application of a biomass-ash-based catalytic ozonation process for the elimination of dyes. The potential of the *Sapindus mukorossi* biosorbent was previously shown for wastewater containing Reactive Red 214 (*C_0_* = 20–80 mg/L) decolorization via the electro-coagulation/biosorption process (the total organic carbon removal efficiency was *%R_TOC_* = 99.56% (*C_0_* = 50 mg/L), *%R_TOC_* = 99.9% (*C_0_* = 20 mg/L), 91.75% (*C_0_* = 80 mg/L)) with the reaction time = 20 min, current density = 21.7 mA/cm^2^, pH 7, and biosorbent dosage = 8 g/L [37]. In the presented investigations, the primary emphasis was on the application capabilities and efficiency of the iron-loaded dead leaf ash (Fe-DLA) as a catalyst with renewable attributes and economic viability. This assessment pertains specifically to its role within the catalytic ozonation process, and a method was employed for the eradication of persistent dye pollutants such as Reactive Black 5.

The present research focused on the application of a novel catalyst of iron-loaded dead leaves of *Sapindus mukorosi* trees in the heterogeneous catalytic ozonation process for Reactive Black 5 treatment of an aqueous solution. Various parameters, like pH, ozone dose, Fe-DLA dose, initial concentration, TBA effect, and reusability, were taken into account.

The experimental endeavors undertaken in this study yielded noteworthy outcomes in terms of decolorization efficiency. This accomplishment holds significant promise, acting as a foundational stepping-stone toward the potential integration and utilization of Fe-DLA in authentic wastewater treatment scenarios. The combination of Fe-DLA and the catalytic ozonation process presents a potentially groundbreaking approach to address the challenge of removing Reactive Black 5 from contaminated water sources, underscoring the practical application of this catalyst and process combination in the domain of environmental remediation.

## 2. Results and Discussion

### 2.1. Catalyst Characterization

**Figure 1:**
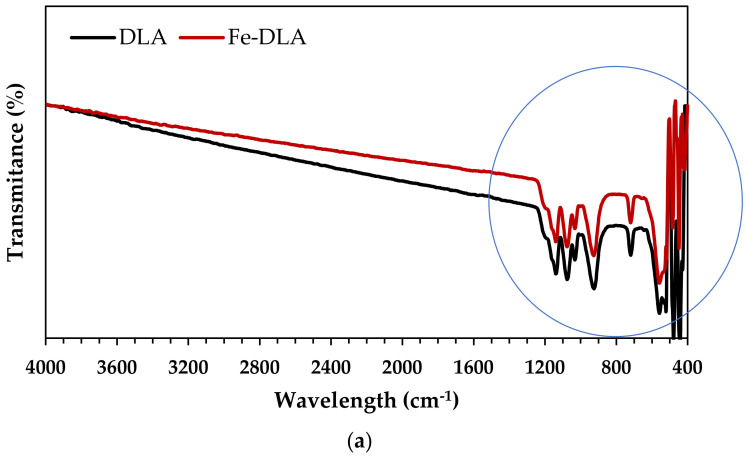

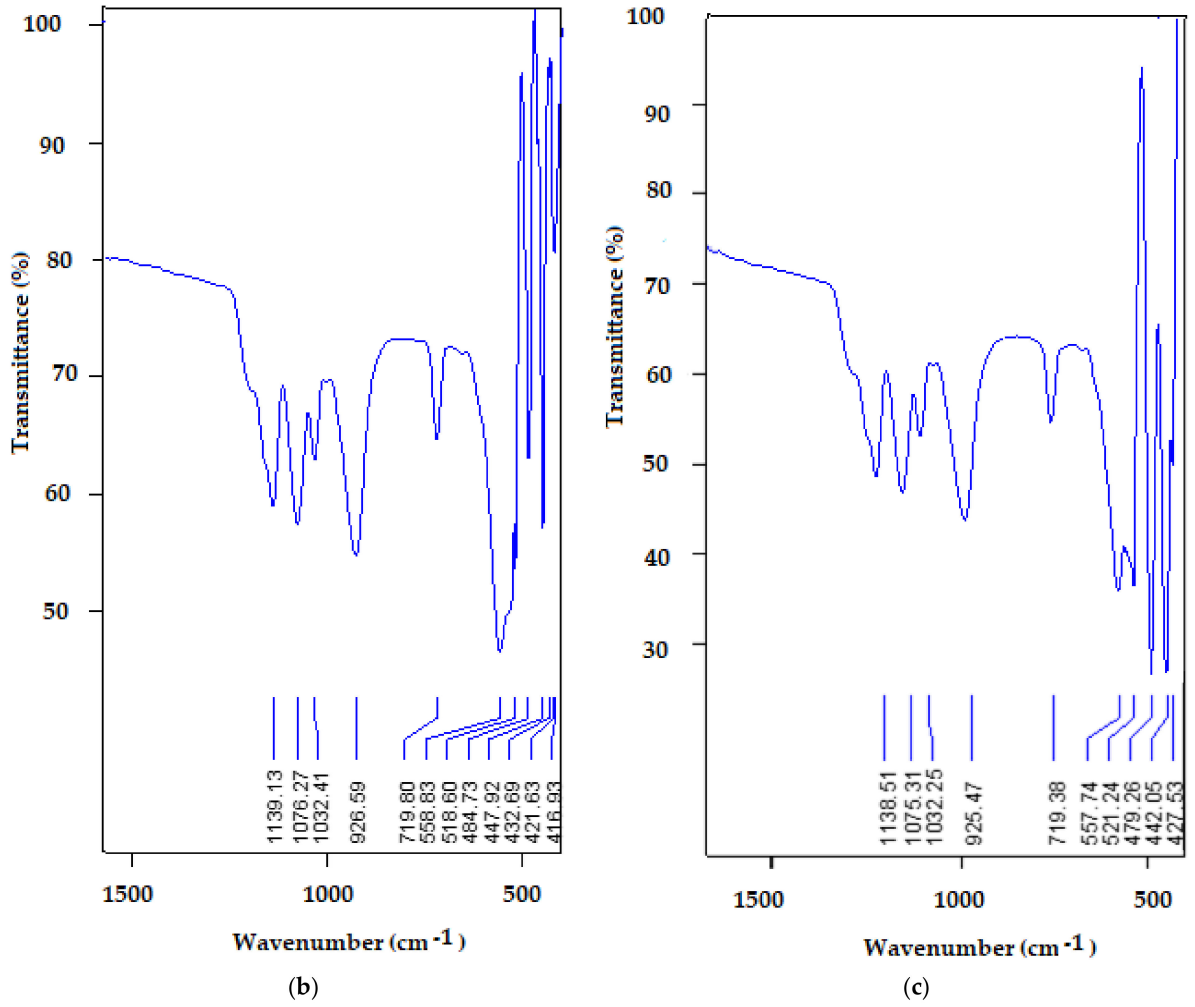
The FTIR spectra of DLA (**a**,**c**) and Fe-DLA catalysts (**a**,**b**).

An Attenuated Total Reflectance Fourier Transform Infrared Spectroscopy (ATR-FTIR) analysis of the dead leaf ash (DLA) and the iron-loaded dead leaf ash (Fe-DLA) was conducted to determine the functional groups present on the catalyst’s surface. The FTIR spectra recorded in the wavelength region from 400 to 4000 cm^−1^ are depicted in Figure 1a, whereas their zoom at wavelengths 400–1600 cm^−1^ are presented in Figure 1c,d.

The DLA and Fe-DLA spectra reveal multiple peaks that can be associated with both stretching (symmetric, asymmetric) and/or bending (scissoring, rocking, wagging, twisting) vibrations. The peaks at 447 cm^−1^ and 484 cm^−1^ represent the O-Si-O bending vibrations of silica. The peaks at 518 cm^−1^ and 558 cm^−1^ represent the Al-Si-O bending vibrations related to the presence of silica. The peaks at 719 cm^−1^ and 926 cm^−1^ represent the Si-O-Si symmetric stretching vibrations. Peaks at 1032 cm^−1^ and 1076 cm^−1^ indicate the Si-O asymmetric stretching vibrations [38,39]. In the previous study [40,41] and the study of various biomass ashes [42], some similar peaks were found.

**Figure 2:**
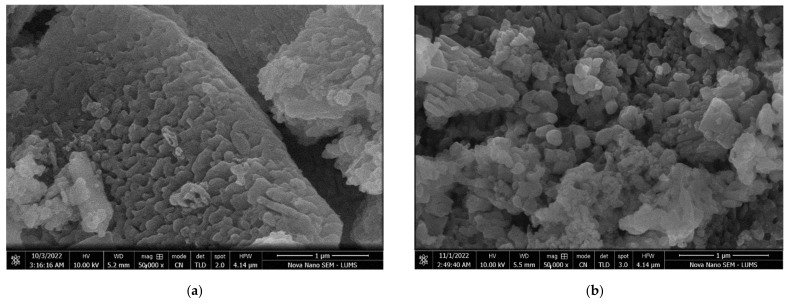
SEM image of (**a**) DLA and (**b**) Fe-DLA.

A surface structural analysis of the DLA and Fe-DLA catalysts was conducted to elucidate the morphological changes that occurred before and after Fe loading. Scanning Electron Microscopic (SEM) images at a magnification of 50,000 were taken, and are shown in Figure 2. The surface morphologies of both the DLA and the iron-doped catalyst look similar. The images clearly depict a porous spongy surface in both cases. The surface contains many cavities, and the structural surface shows a potential dye attachment ability, and the possibility of proceeding with the catalysis. No major visible difference was found in the SEM images of DLA and Fe-DLA.

The elemental analysis of the DLA and Fe-DLA was conducted using an energy-dispersive X-ray spectroscopy (EDX) analysis, which displayed some clear peaks in Fe in the case of Fe-DLA. The EDX spectra of DLA and Fe-DLA are presented in Figure S2, whereas the elemental analysis is presented in Table 1.

The EDX results revealed the presence of 1.1% iron in the DLA catalyst. The EDX analysis of DLA showed its composition, and C (8.39%), O (30.68%), Si (1.10%), P (35.9%), Ca (21.71%), and Fe (1.1%) were found. The EDX spectrum of Fe-DLA showed the presence of 17.01% iron, confirming the loading of iron on the DLA. The amount of loaded Fe is satisfactory when using the standard method if compared with the previous works in the literature and can provide an efficient base for the conversion of O_3_ to OH radicals [43].

The surface area and pore size of the prepared catalyst was found using the Bruner–Emmet–Teller (BET) method (Table 2).

**Figure 3:**
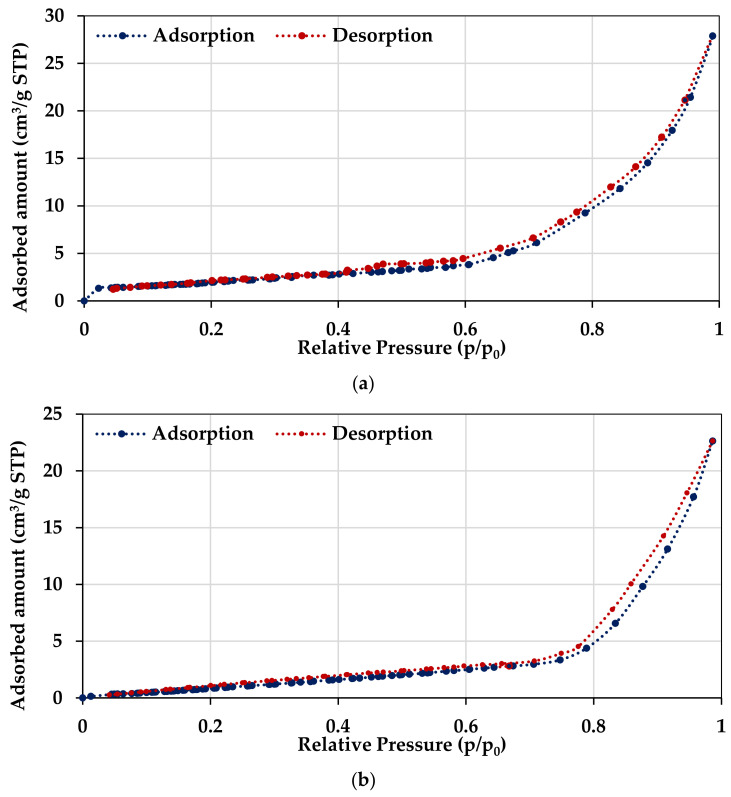

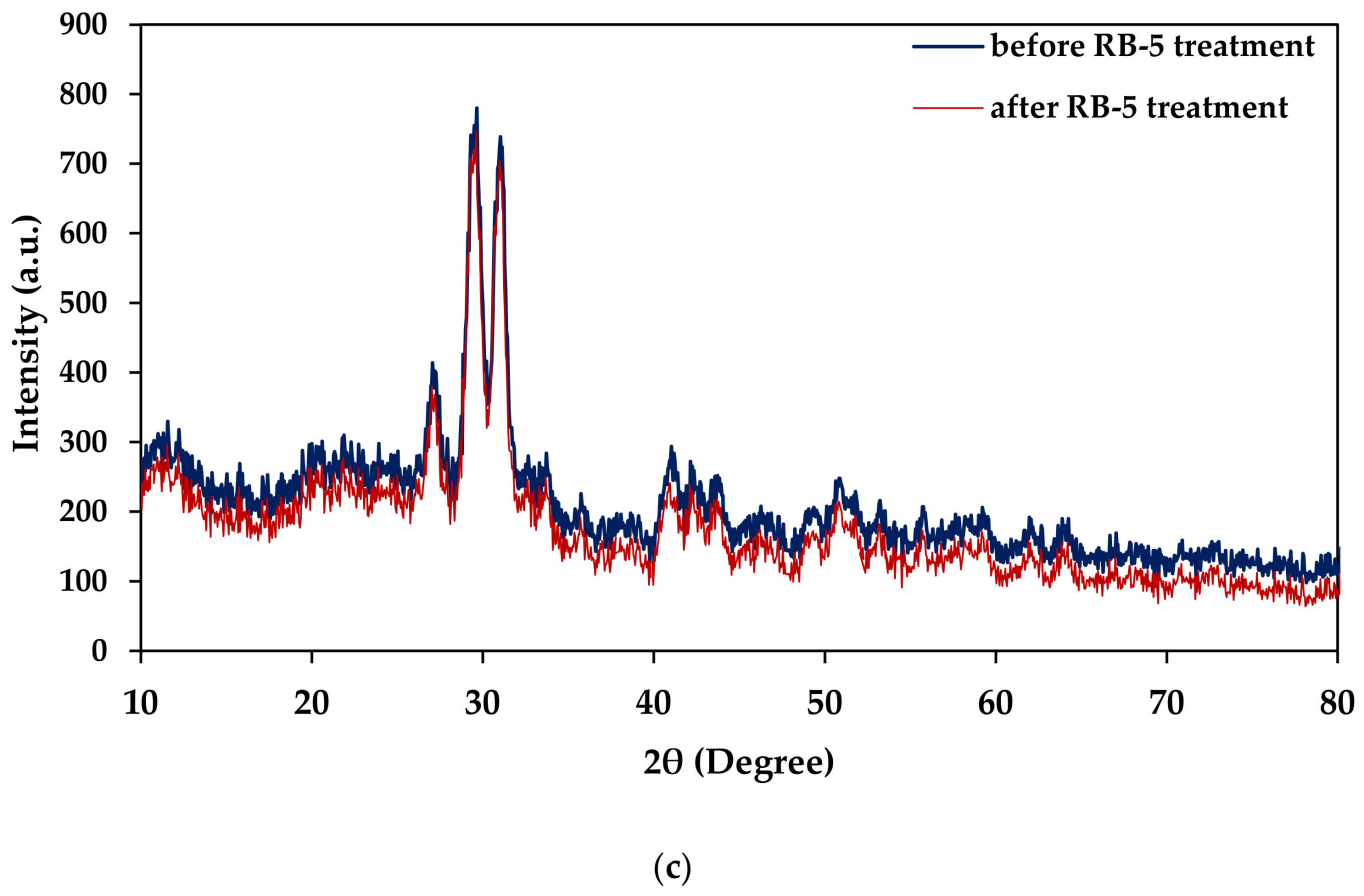
(**a**,**b**) The low-temperature nitrogen adsorption–desorption isotherms and (**c**) the XRD spectra for (**a**) DLA, (**b**) Fe-DLA, and (**c**) Fe-DLA catalysts before and after the treatment of wastewater containing RB-5 dye.

The specific surface area was not very large, because it assumes a small value of 14.20 m^2^/g, whereas the mean pore size was equal to 1.75 nm for DLA. The nitrogen adsorption–desorption hysteresis loop shapes were employed to determine the specific pore structures and explore the textural properties of the DLA and Fe-DLA catalysts. The low-temperature nitrogen adsorption–desorption isotherms for the DLA and Fe-DLA catalysts are depicted in Figure 3a,b. The DLA catalyst has a higher adsorption capacity than Fe-DLA. The adsorption amount at a *p/p*_0_ of around 0.995 is equal to 27.89 cm^3^/g for the DLA catalyst and 22.63 cm^3^/g for the Fe-DLA catalyst. The N_2_ adsorption content in the DLA catalyst in the low-relative-pressure ranges (<0.02) is around two times higher compared to the Fe-DLA catalyst. According to the IUPAC classification, which distinguished the six types of isotherms (I–VI), the shape of low-temperature nitrogen adsorption/desorption isotherms is characteristic of type II (nonporous or microporous), which behaves like a porous media containing macropores and mesopores. The obtained average pore size values confirm the presence of micropores (<2 nm) in the catalysts’ structure. For these samples, N_2_ molecules first diffuse into the micropore under low pressure, then, with the increase in relative pressure, are adsorbed in a monolayer state and in subsequent layers as a condensation state [44]. The low-temperature N_2_ adsorption isotherms show weaker hysteresis loop effects, and the hysteresis phenomenon is usually associated with capillary condensation in mesopore structures. The obtained hysteresis loops belong to the H3 type, indicating that slit-shaped pores are expected in these samples [45].

The results showed that after Fe was loaded on DLA, its surface area decreased from 14.2 m^2^/g to 11.3 m^2^/g and a slight increase could be seen in the pore size, from 1.75 nm to 1.81 nm. The BET surface area of the DLA catalyst decreased after the Fe, perhaps due to surface capping by Fe(II) metal ions, as revealed by previous studies [42,43]. The Fe-loaded catalyst plays an important role in the catalytic oxidation process. Previous findings [46,47] indicate that Fe may interact with aqueous ozone, leading to the production of OH radicals. OH radical formation is important in such an oxidation process to achieve significant dye removal. Therefore, the Fe loading enhances the catalytic activity in this type of heterogeneous catalytic ozonation process.

The XRD spectra of the Fe-DLA catalyst before and after its addition to wastewater containing the RB-5 dye treatment are shown in Figure 3c. The spectra before treatment indicate the amorphous nature of the material. A major peak traced at 2 theta of 29.66 represents the silica phase, which is present in the ash as a major constituent. The Fe^2+^ peak is traced at 2 theta of 41.03, which indicates the presence of iron, whose valency did not change after its addition to wastewater containing the RB-5 treatment. The peaks obtained at 31.03 and 27.22 degrees correspond to other major constituents in ash, such as Ca and P, respectively [48,49].

The points of zero charge (*pH_pzc_*) in DLA and Fe-DLA achieved using the mass titration method were found to be 8.0 ± 0.2 and 7.1 ± 0.1, respectively.

### 2.2. Parameters Effect Study

#### 2.2.1. Effect of Ozone Dose in the Single Ozonation Process

**Figure 4:**
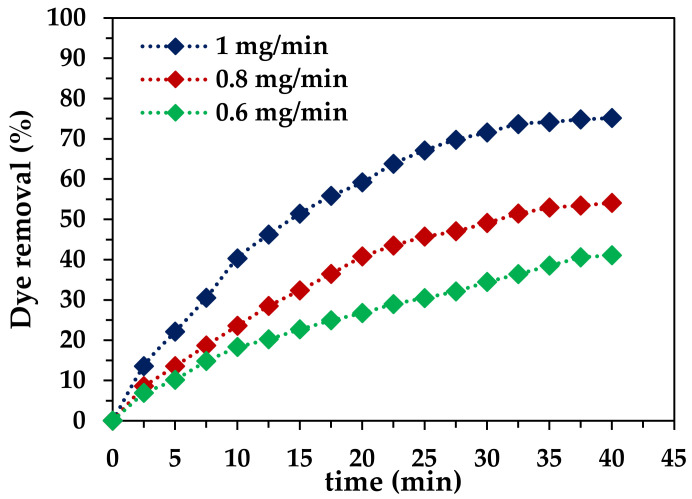
Effect of ozone dose in the simple ozonation process (pH 5.6, *T* = 25 °C, *C_0_* = 50 mg/L, *V* (volume) = 1.0 L, catalyst dose = 0 g/L).

To set a suitable ozone dose in the system, the ozone dose was studied using a single ozonation process for RB-5 removal. The oxidizing power of O_3_ makes it an efficient oxidant; hence, it can be used as an effective oxidant for the mineralization of dyes. The ozone dose varied in the range from 0.6 mg/min to 1.0 mg/min and decolorization wastewater containing RB-5 was analyzed. The results (Figure 4) clearly show an effective increase in color removal with an increase in the ozone dose, and maximum removal was achieved at 1.0 mg/min ozone dose. After 40 min of treatment, 41.08% dye removal was achieved at 6 mg/min dose, while removal yield being 54.08% and 75.13% were achieved at 0.8 mg/min and 1.0 mg/min, respectively. At an alkaline pH, a high ozone dose causes the rapid generation of quickly oxidizing OH radicals, which enhances the mineralization rate of the organic dye molecules, thereby accelerating the rate of removal [43,50,51].

#### 2.2.2. Effect of pH

**Figure 5:**
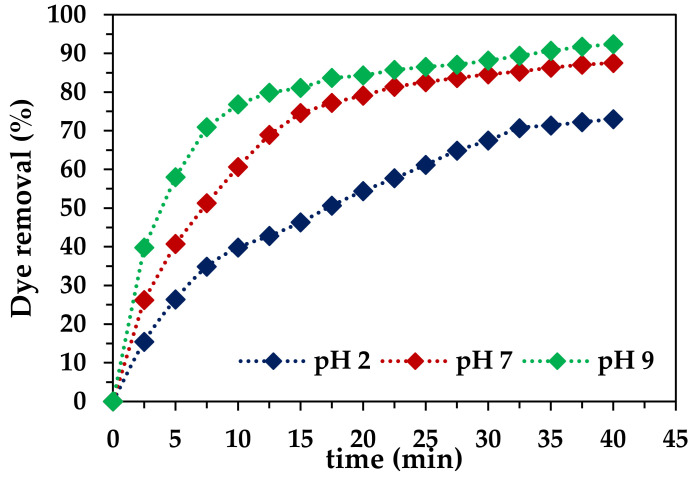
Effect of pH on dye removal by O_3_ (*t*(time) = 40 min, pH 2, 7, 9, ozone dose = 1.0 mg/min; *T* = 25 °C; *C_0_* = 50 mg/L, *V* = 1.0 L, catalyst dose = 0 g/L).

The pH of the dye solution was adjusted at 2, 7, and 9 using 0.1 mol/L HCl and 0.1 mol/L NaOH to study its effect on the ozonation process. The effect of pH on the percentage removal of RB-5 dye is depicted in Figure 5.

In this simple ozonation, the presented results clearly show an increase in the removal of RB-5 dye as the pH shifts from the acidic to basic. At pH 2, the maximum removal was achieved at 40 min of treatment and was equal to 72.9%. The graph is not very steep at any stage of the treatment, and displays a slow and steady process of dye removal. This is because only the molecular O_3_ reacts in the acidic medium, and RB-5 molecules, being complex molecules, do not degrade abruptly [11]. In a neutral medium, the rate of dye removal was higher because, in the presence of hydroxyl ions, some of the O_3_ is converted into hydroxyl radicals. Such radicals have a high (2.8 V) oxidation potential to degrade dye molecules, enhancing the rate, and after 40 min of treatment, 87.5% of the dye was removed. After a further increase in the pH to 9, the concentration of hydroxyl ions in the basic pH increases, and the conversion of molecular O_3_ into OH radicals with a much higher oxidation potential than O_3_ was observed [52]. Consequently, the utilization of an alkaline medium results in significant enhanceent in the overall efficiency of wastewater treatment. Additionally, within a remarkably short treatment time, equal to 15 min, a substantial elimination of approximately 81.0% of the dye was achieved. Furthermore, extending the treatment duration to 40 min yielded even more satisfactory results, with a substantial RB-5 removal of 92.4%. These results indicate the significant role of the alkaline medium in wastewater treatment containing dye, and highlight its potential for rapid and efficient dye elimination [52]. The reason for the better removal efficiency in a basic medium is due to the presence of hydroxyl radicals in the solution, and the maximum removal was achieved at pH 9. Previous studies have shown that the HCOP has maximum activity at a pH close to that of wastewater [25].

#### 2.2.3. Effect of Fe-DLA Dose

**Figure 6:**
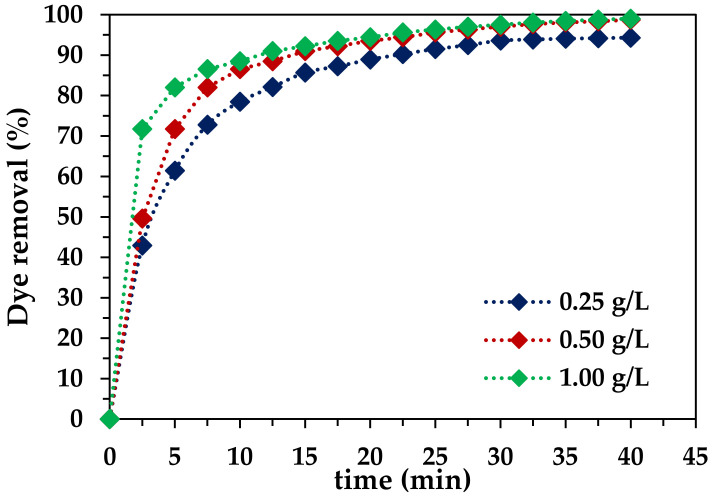
Effect of catalyst dose on HCOP (*t* = 40 min, pH 9, *T* = 25 °C, *C_0_* = 50 mg/L, ozone dose = 1.0 mg/min, *V* = 1.0 L, catalyst dose = 0.25–1.0 g/L).

At the selected ozone dose and pH, the catalyst dose was varied, at 0.25 g/L 0.50 g/L, and 1.0 g/L, to elucidate its effect on the decolorization of wastewater containing RB-5 dye (Figure 6). When 0.25 g/L of the Fe-DLA catalyst was used, the removal rate was too slow in the first 10 min of treatment, i.e., 78.48%, and after 40 min, 94.31% of RB-5 dye was removed. When using a 0.5 g/L dose of the catalyst, the dye removal efficiency was 86.53% after 10 min and 98.75% after 40 min, whereas using of 1.0 g/L of the catalyst led to a dye removal yield of 90.99% (10 min) and 99.01% (40 min). For that specific volume, concentration, and ozone dose, the results were not as good with a 0.25 g/L catalyst dose; upon increasing the concentration to 1.0 g/L, the removal rate increased. Hence, 0.5 g/L was selected as a comparatively better dose, considering both the cost and efficiency of the treatment process. The augmentation in the quantity of the catalyst introduced to the system corresponds to the creation of a more extensive platform, offering an increased surface area. This augmented surface area facilitates the accelerated attachment of dye molecules. As a result, the subsequent interaction with hydroxyl (OH) radicals is expedited. This heightened interaction, in turn, serves to bolster the pace at which dye molecules undergo mineralization. In essence, the escalation in catalyst dose plays a pivotal role in fostering a more conducive environment for the swift degradation of dye molecules, ultimately contributing to the accelerated and efficient mineralization of the dye [43,50].

#### 2.2.4. Effect of RB-5 Initial Concentration

**Figure 7:**
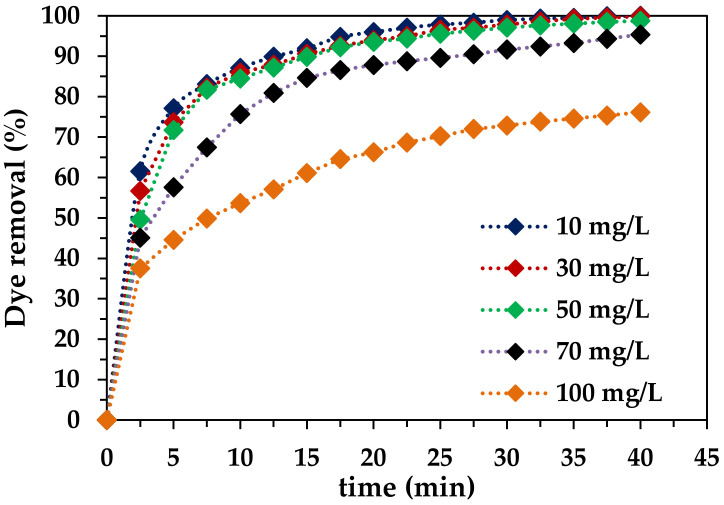
Effect of initial RB-5 concentration on HCOP (*t* = 40 min, pH 9, *T* = 25 °C, *C_0_* = 10–100 mg/L, ozone dose = 1.0 mg/min, *V* = 1.0 L, catalyst dose = 0.50 g/L).

The initial concentration of RB-5 was altered in the range of 10–90 mg/L to check its effect on the efficiency of pollutant removal using the Fe-DLA/O_3_ process. The results depicted in Figure 7 indicate that with the increase in the initial concentration of dye, the removal efficiency decreases. After 40 min of treatment, the highest RB-5 removal, equal to 99.95%, was achieved at an initial concentration of 10 mg/L and the lowest removal of 76.53% was observed at 90 mg/L. RB-5 removal efficiencies of 99.8%, 98.8%, and 95.3% were achieved at 30, 50, and 70 mg/L, respectively. Figure 7 shows that the curves of dye removal *(*%) vs. *t* are steeper in the first 10–15 min of treatment; after that, *%R* increases slightly over time [43]. The findings of the study unveil a discernible disparity in the pace of dye removal when using higher and lower concentrations of RB-5. Notably, the removal process appears to exhibit a relatively sluggish performance at elevated RB-5 concentrations in contrast to lower concentrations. This observed divergence in behavior could potentially stem from a limitation in the availability of active sites on the catalyst surface at higher dye concentrations. This scarcity of reactive sites may impede the swift interaction between the dye molecules and the catalyst, consequently leading to a decelerated removal rate [25].

#### 2.2.5. Process Comparison Study

**Figure 8:**
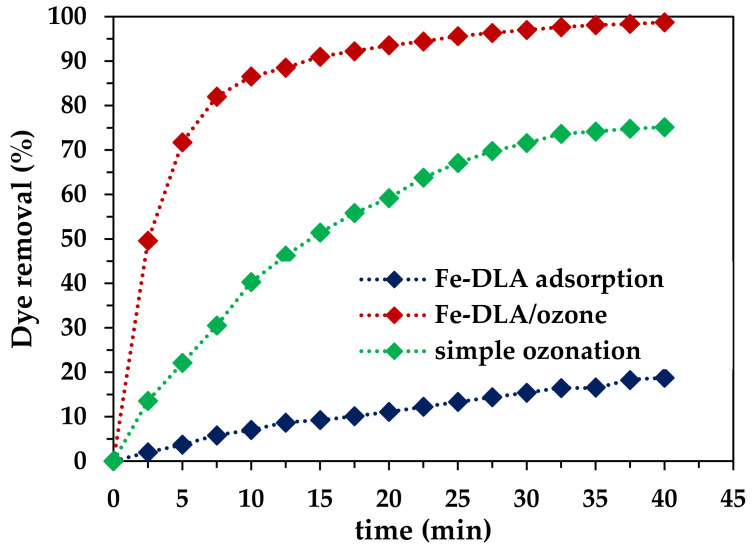
Comparative study of dye removal by various processes (*t* = 40 min, pH 9, *T* = 25 °C, *C_0_* = 50 mg/L, ozone dose = 1.0 mg/min, *V* = 1.0 L, catalyst dose = 0.50 g/L).

A comparative study of Fe-DLA adsorption, simple ozonation (O_3_), and catalytic ozonation (Fe-DLA/O_3_) was performed at the following optimum parameters: pH 9, ozone dose 1.0 mg/min, catalyst dose 0.5 g/L, and 50 mg/L RB-5. The results are shown in Figure 8. The process’ efficiency could be arranged in the following order: Fe-DLA/O_3_ > O_3_ > Fe-DLA adsorption. Simple ozonation at optimum parameters removed 92.38% of the dye, and when the dye solution was kept for adsorption on the Fe-DLA catalyst with continuous stirring with a magnetic stirrer, 18.99% of the RB-5 dye was removed after 40 min of treatment [42]. For comparison, the Fe-DLA/O_3_ process was the most efficient of the three processes, removing 99.89% of the dye in 40 min. Most importantly, in the case of HCOP, the curve is initially steeper, showing that the rate of conversion is much higher compared to the other processes [20]. In comparison, the percent of RB-5 removal in the first 10 min of treatment with the Fe-DLA/O_3_ process was 86.53%; in treatment with simple O_3_, it was 76.82%; and in the Fe-DLA adsorption treatment, it was just 7.03%. The rapid rate of RB-5 removal and overall higher efficiency clearly suggest that HCOP using the Fe-DLA catalyst may be an efficient treatment method for the continuous removal of effluents from textile wastewater containing dyes, and may be helpful in recycling and conserving water [19,24,25].

Previous research works [53,54] indicate the effective application of leaf-based ash as a low-cost biosorbent for the elimination of textile dyes. Most of the studies conducted on biomass ash involve the adsorption of dyes; for example, Duan et al. [53] investigated the performance of neem leaf ash as an economical green adsorbent for the treatment of various dyes. A stronger adsorption of cationic dyes was achieved, with an adsorption yield of up to 91.98%, and a highly consistent reusability performance for up to five cycles. Alam et al. [54] studied the removal of Methylene Blue on indigenous biosorbents such as banana leaf ash and rice husk ash. A high removal of up to 93.75% of the dye was achieved by banana leaf ash under optimum conditions. These studies revealed that leaf ash has potential in Methylene Blue removal.

However, limited research has been reported on the application of a biomass-ash-based catalytic ozonation process for the elimination of dyes. The present study is the first investigation on the use of dead leaf ash as a renewable, low-cost catalyst in the catalytic ozonation process for the removal of RB-5. Babar et al. [55] investigated the removal of Methylene Blue by iron-loaded, biochar-based heterogeneous catalytic ozonation. The research showed a high catalytic performance of biochar ash, achieving 95.82% dye elimination at a higher catalyst dose of 5 g/L, as compared to the present study, which showed a high ash performance, with 98.76% dye removal, at even a low dose of 0.5 g/L. Asgari et al. [56] reported the elimination of RB-5 using the catalytic ozonation process on modified bone char ash decorated with MgO–FeNO_3_. Up to 90% of dye was degraded at a low catalyst dose of 0.1 g/L. As compared to the present study, the optimum results were achieved at pH 10, and the dead leaves’ abundant availability makes the catalyst readily available compared to bones. Therefore, the dead-leaf-based catalyst offers an eco-friendly low-cost solution to dye-contaminated wastewater as compared to the materials studied in the literature.

#### 2.2.6. COD Removal Study

**Figure 9:**
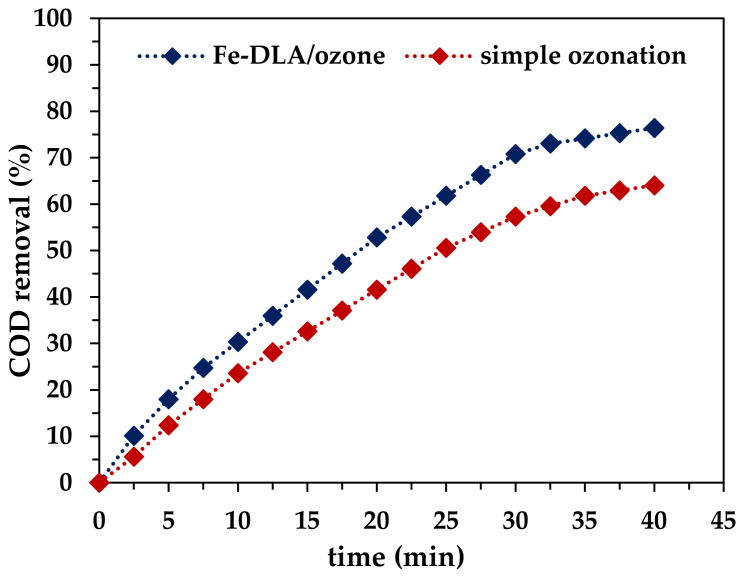
COD removal by HCOP at optimum parameters (*t* = 40 min, pH 9, *T* = 25 °C, *C_0_* = 50 mg/L, ozone dose = 1.0 mg/min and 0 mg/min, *V* = 1.0 L, catalyst dose = 0.50 g/L).

COD concentration before and after treatment was studied using the standard vial reagent methods and simple ozonation and catalytic ozonation. The results (Figure 9) show a 12.0% increase in the removal of COD in the case of Fe-DLA/O_3_ catalytic ozonation in comparison to the simple ozonation process. The addition of the catalyst facilitates the generation of hydroxyl radicals, which mineralize the contaminant more efficiently compared to the simple ozone method, resulting in more COD removal in the defined time. The COD removal efficiency reached 76.44% in the case of the Fe-DLA/O_3_ process, whereas the removal efficiency for simple ozonation was equal to 64.04% after 40 min of treatment [49,57,58].

#### 2.2.7. TBA Effect

**Figure 10:**
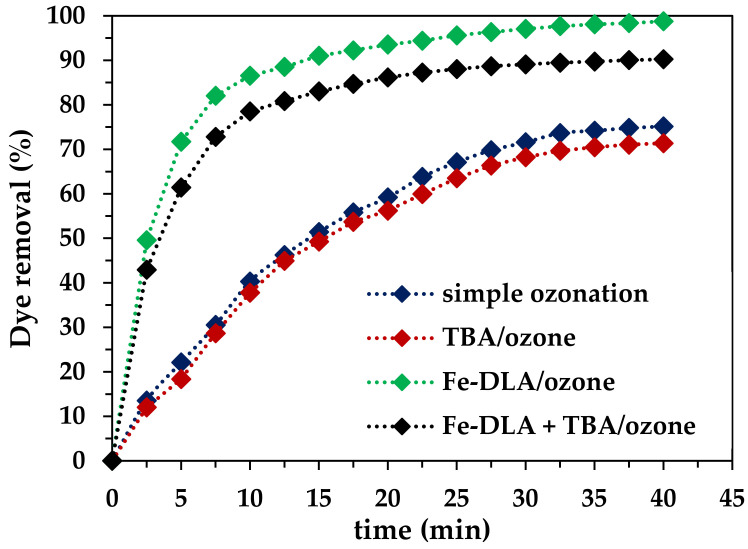
TBA effect on dye removal (*t* = 40 min, pH 9, *T* = 25 °C, *C_0_* = 50 mg/L, ozone dose = 1.0 mg/min, *V* = 1.0 L, catalyst dose = 0.50 g/L, TBA = 20 mg/L).

Catalytic ozonation aims to generate OH radicals by breaking down the O_3_ on the catalyst surface. Tert-butyl alcohol (TBA) was used as a hydroxyl radical scavenger to confirm the generation of OH radicals in this process. The TBA effect on the RB-5 removal efficiency was studied under optimum conditions. The plot in Figure 10 clearly depicts the greater decrease found in the removal efficiency when the TBA was used, and the reduction yield is higher in the case of the Fe-DLA/O_3_ process compared to the simple ozonation process. Additionally, the dye removal efficiency decreased from 75.13% to 71.35% and from 98.74% to 90.22% for the simple O_3_ and HCOP, respectively, when using the Fe-DLA processes. The considerable decrease in removal in the case of the catalytic process clearly suggests the generation of OH radicals in the Fe-DLA/O_3_ process.

#### 2.2.8. Catalyst Reusability Study

**Figure 11:**
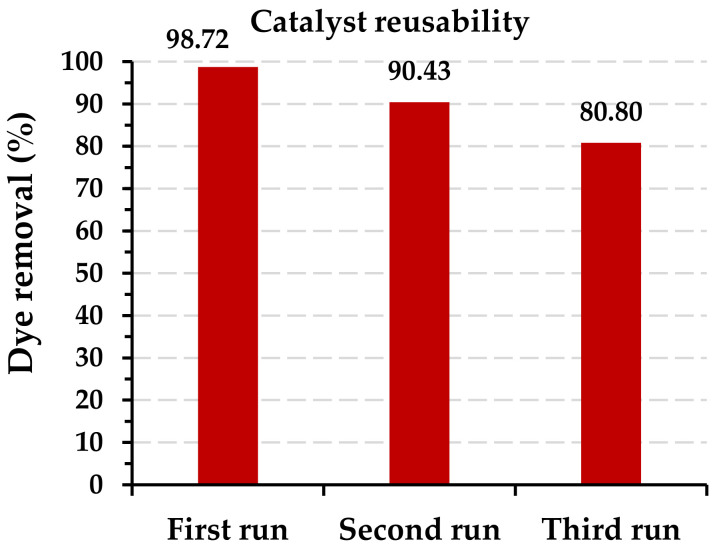
Catalyst reusability study (*t* = 40 min, pH 9, *T* = 25 °C, *C_0_* = 50 mg/L, ozone dose = 1.0 mg/min, *V* = 1.0 L, catalyst dose = 0.50 g/L).

The catalyst’s performance and cost-effectiveness are concluded in the reusability study. The Fe-DLA catalyst was reused for up to three runs consecutively in the Fe-DLA/O_3_ process, and Figure 11 shows the results. For the second run, the catalyst was washed with distilled water and reused. The removal efficiency dropped in the following runs, from 98.72% (first run), through 90.42% (second run), to 80.80% (third run). Despite of the catalyst efficiency dropped the catalyst has still ability for the dye removal. This reduction in the catalyst’s performance may be attributed to the dye molecules becoming attached to the pores and surface of the catalyst, and the iron leaching out during the treatment. The reusability study shows that the catalyst is stable enough to use for a few cycles at an industrial scale.

Each experimental run was conducted by loading Fe-DLA via the RB-5 dye, whose concentration in wastewaters was 5 mg/L. At the end of each run, the catalyst was recovered by filtration, with an average recovery of 98.0 ± 0.3%. Iron leaching tests were conducted, and the average iron leaching rate was determined to be 0.091 ± 0.01 mg/L, which was not considered significant.

#### 2.2.9. Proposed Mechanism

**Figure 12:**
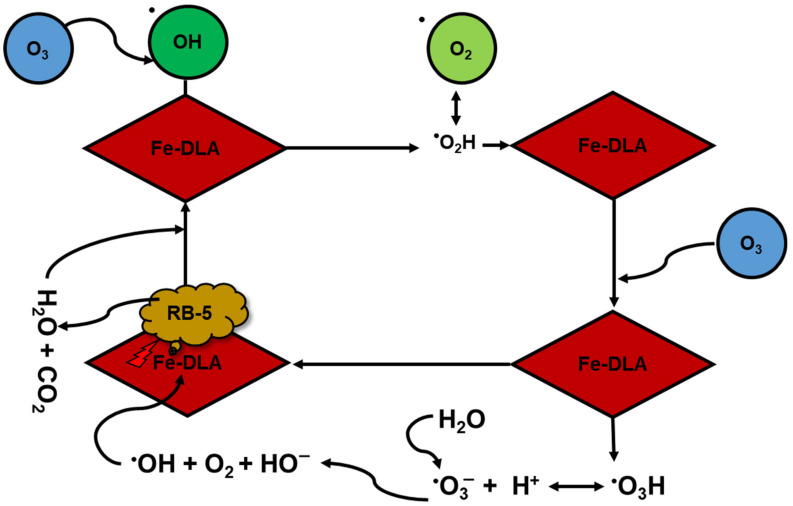
Proposed mechanism of Fe-DLA catalytic ozonation for RB-5 removal.

The proposed mechanism of RB-5 removal via Fe-DLA catalytic ozonation is shown in Figure 12. At pH 9, the OH radicals are readily generated from ozone and adopt their positions on the surface platform of the Fe-DLA catalyst. It is hypothesized that aqueous ozone interactions with surface hydroxyl groups on Fe-DLA lead to the generation of superoxide ion radicals [46,47]. These radicals further interact with the aqueous ozone to generate faster oxidant hydroxyl radicals, as shown in Figure 12. The hydroxyl radicals rapidly attack the dye molecules and mineralize them. The hypothesis concerning the radical-based mechanism in this study seems to be possible due to the TBA effect shown in Figure 10, but the OH radicals’ dominant role could not be definitely confirmed. The process comparison study (Figure 8) indicates both a slight adsorption effect of the catalyst and the involvement of hydroxyl radicals in the dye’s degradation [46,47].

## 3. Materials and Methods

### 3.1. Material and Reagents

The Reactive Black 5 (RB-5) was supplied by Sigma Aldrich, Gillingham, UK (Table 3). Various chemicals used for the Fe-DLA preparation, including FeSO_4_, HNO_3_, HCl, NaOH, Na_2_S_2_O_7_, KI, NaHCO_3_, and Na_2_CO_3_, were provided by Merck, Darmstadt, Germany. All chemicals used were of analytical grade, and were used without further purification. The standard solutions were prepared through standard methods.

### 3.2. Preparation of Fe-DLA

The dead waste leaves of *Sapindus mukorossi* were collected from Begum pora, Lahore, and washed multiple times with distillated water to remove dirt and attached contaminants. The washing procedure was repeated until a constant pH was observed. The dead leaves were converted to powdered biomass using the method adopted from [37,59].

The prepared biomass powder was placed in a furnace at 650 °C for conversion into dead leaf ash (DLA). The formed DLA was then immersed in 0.1 mol/L HNO_3_ for more than 24 h, and then filtered through suction filtration and washed with deionized water until the pH of the water was neutralized. Then, it was kept in the oven at 110 °C overnight [13]. Then, the 0.176 mol/L solution of FeSO_4_ was prepared by dissolving 4.96 g of FeSO_4_ in 100 mL of deionized water. The impregnation method was used to load iron on the DLA, for which 6.0 g of the DLA was taken and dissolved in 30 mL of the prepared solution. The solution was then kept on a hot plate at 60 °C and stirred for 6 h until the water evaporated. After that, it was kept in the oven at 100 °C for 6 h. The obtained ash was then washed with deionized water and dried.

### 3.3. Catalytic Ozonation Experiments

**Figure 13:**
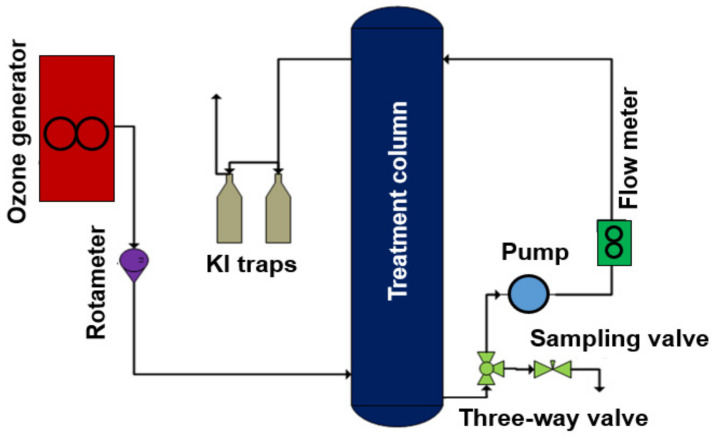
Experimental setup of the heterogeneous catalytic ozonation process (HCOP).

The stock solution of Reactive Black 5 dye of 1000 mg/L was meticulously concocted by solubilizing a proper amount of dye in the distilled water. After that, this solution was applied in the preparation of working solutions of various concentrations of RB-5 dye, using the standard dilution method. The setup for RB-5 removal using the HPOC method is depicted in Figure 13.

This experimental scheme provides an essential visual aid for comprehending the configuration and arrangement of the apparatus, enhancing the clarity and understanding of the experimental procedure employed in this study. A glass column with a height of 914.5 mm and an inner diameter of 63.4 mm was used as a reactor. Ozone was generated from atmospheric air using a locally manufactured ozone generator. Two gas wash bottles were used, containing 250 mL of 2.0% KI solutions dissolved in deionized water. A total of 1.0 L of the dye solution was put into the reaction column for each run of a different initial concentration, with proper parameter adjustments. Varying amounts of the catalyst were introduced in the column from the top by removing the lid. Ozone was introduced into the column through a porous diffuser from the bottom of the column. A recirculation pump (Iwaki, Japan, Model: MD-6K) with a capacity of up to 3 L/min was attached to the bottom of the column. The solution was recirculated in the column through the pump at a constant rate of 0.5 L/min, controlled by the flow control valve on the flowmeter. This recirculation not only enhances the contact between the ozone and dye solution via turbulence, but also reduces the amount of unreacted ozone that escapes to KI traps via direct interactions. The ozone produced by the ozone generator was sparged into the solution at the bottom of the ozonation column using a ceramic sparger. The ozone, in the form of tiny bubbles with an average size of 1–3 mm, was continuously injected into the solution during the reaction period. The bubbles caused a high level of turbulence and the fast degradation of the dye molecules, while the off gas was engaged in KI traps. A radical scavenger study was conducted using Tert-Butyl Alcohol (TBA) as a hydroxyl radical scavenger. For scavenger studies, at the start of the experimental run, after loading the catalyst dose and setting the ozone flow, 20 mg/L of TBA was added to the reaction column and samples were withdrawn and analyzed after fixed time intervals. Samples collected from the sample collection valve at different intervals were quenched with 0.025 mol/L Na_2_CO_3_ to remove the residual O_3_, given 10 min of settling time, filtered, and finally analyzed to obtain the RB-5 concentration [13,57].

### 3.4. Analytical Methods

The concentration of dye in the solutions after treatment was obtained with the absorbance analysis using the Perkin Emler lambda 35 double-beam UV-Vis Spectrophotometer. The absorbance of all samples was measured at a maximum absorbance wavelength λ_max_ = 596 nm [52].

The ozone dose was measured using the standard iodometric method. The ozone produced from the ozone generator was captured in 2.0% of KI solution in distilled water, and then quenched by the addition of 5 mL of 1 mol/L H_2_SO_4_ solution. The solution was then titrated with 0.0025 mol/L Na_2_S_2_O_3_, and starch was used as an indicator. Using the runtime of the ozone generator, the total volume of sodium thiosulphate consumed, and its concentration, the ozone concentration was found.

The Chemical Oxidation Demand (COD) of the samples extracted under optimum conditions was measured with the help of standard digestion methods using the COD vials, and the percent removal was measured by relating the decrease in COD with the initial COD of the 50 mg/L aqueous solution. The initial COD of the 50 mg/L RB-5 solution was determined to be 135 mg/L.

### 3.5. Catalyst Characterization Techniques

The surface morphology of the prepared novel catalyst was studied using Scanning Electron Microscopy (SEM), and its elemental composition was found via Energy-Dispersive X-ray Spectroscopy (EDX) using the Nova NanoSEM 450 analyzer. Images were captured with the electron beam at an acceleration voltage of 10 kV. The surface area and pore size were analyzed with the Brunauer–Emmet–Teller (BET) method using the Micrometrics-ASAP-2020 analyzer. Samples were degassed under vacuum at 350 °C for 8 h. The 99.99% pure nitrogen adsorption–desorption isotherms were measured at 77 K. Functional groups present in the Fe-DLA were analyzed by FTIR-ATR spectrum using the FTIR Spectrometer Alpha-E, Bruker, in the wavelength region from 400 to 4000 cm^−1^ at a resolution of 2 cm^−1^, using diamond single-reflection attenuated total reflectance. The X-Ray diffraction analysis (XRD) was applied to evaluate the crystallinity and chemical composition of the solid catalyst. The XRD spectrum of the Fe-DLA catalyst was analyzed using the Bruker D2 Phraser X-ray diffractometer in the 2θ range of 10–80, with X-ray generation at 30 kV and 10 mA.

## 4. Conclusions

The DLA-based catalytic ozonation technique has demonstrated remarkable efficiency in degrading toxic Reactive Black 5 molecules. The HPOC results show substantial degradation rates of RB-5 (reaction time of 40 min) and impressive removal percentages (99.89%), highlighting the efficiency of this approach. The first application of low-cost dead-leaf-ash-based catalyst Fe-DLA in HCOP for RB-5 removal in an aqueous solution, in comparison with other methods, such as simple ozonation and Fe-DLA adsorption, shows the significant advantages of the HPOC method over other methods, as follows: Fe-DLA/O_3_ > O_3_ > Fe-DLA adsorption. At optimal conditions, such as 50 mg RB-5/L, pH 9, an ozone dose of 1.0 mg/min, and the Fe-DLA dose of 0.5 g/L, up to 99% of RB-5 was removed, with COD removal being 76.44% in the Fe-DLA/O_3_ process. The addition of a TBA scavenger to the system caused a slight reduction in dye removal in the studied time, which proved that OH radicals participate in the degradation of RB-5. By effectively tackling the removal of toxic Reactive Black 5, this technique shows the potential to contribute significantly to the sustainable treatment of textile-dye-contaminated wastewater, ultimately safeguarding our environment and human well-being. As we continue to refine and expand this innovative approach, we remain committed to the pursuit of cleaner and healthier water resources for future generations.

## Figures and Tables

**Table 1:** EDX composition of DLA and Fe-DLA catalysts applied in the RB-5 wastewater treatment.

Element	Weight (%)	
	DLA	Fe-DLA
C	8.39	5.79
O	30.68	29.83
Si	1.10	1.00
P	35.90	27.7
Ca	21.71	18.37
Fe	1.10	17.01
Cu	1.12	0.30

**Table 2:** Catalyst surface characteristics.

Material	Surface Area	Pore Size
DLA	14.20 m^2^/g	1.75 nm
Fe-DLA	11.30 m^2^/g	1.81 nm

**Table 3:** General characteristics of Reactive Black 5.

IUPAC name	4-amino-5-hydroxy-3,6-bis((4-((2-(sulfooxy) ethyl)sulfonyl) phenyl)azo)-2,7-naphthalenedisulfonic acid tetrasodium salt
Synonyms	C.I. Reactive Black 5, Reactive Black 5, Reactive Black B, Remazol Black 5, Remazol Black B, Drimaren Black R/K-3B
Chemical structure 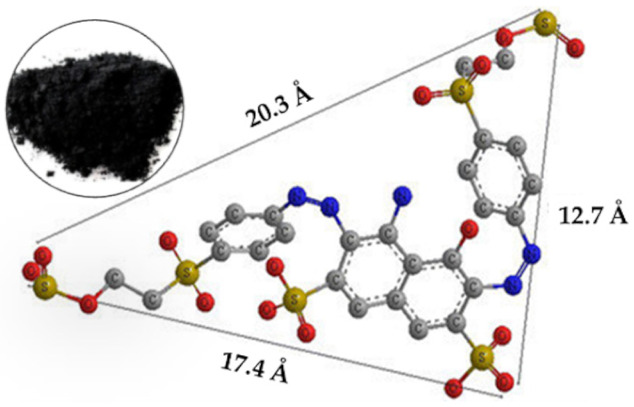	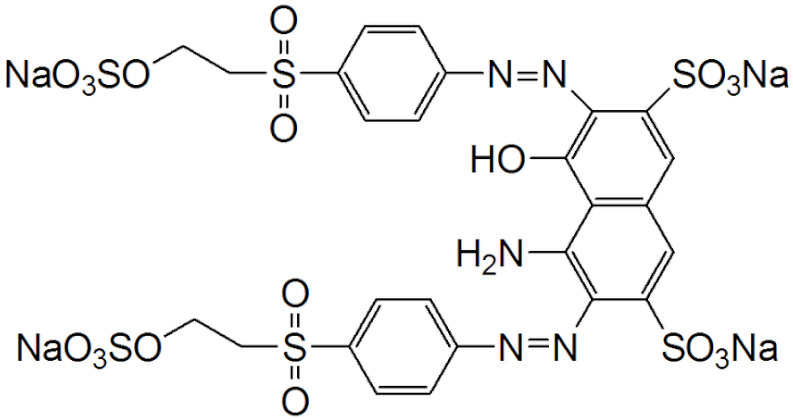
Molecular formula	C_26_H_21_N_5_Na_4_O_19_S_6_
Molecular weight	991.82 g/mol
Dye content	≥50%
Color index (C.I.) name	C.I. Reactive Black 5
Color index (C.I.) number	C.I. 20505
Chemical class	Azo
Application class	Cotton
CAS register number	17095-24-8
λ_max_	596 nm

## Data Availability

Data is contained within the article or Supplementary Materials.

## Supplementary Materials

The following supporting information can be downloaded at: https://www.mdpi.com/article/10.3390/molecules29040836/s1, Figure S1. Comparison of advantageous and disadvantageous of dyes containing wastewater decolorization; Figure S2. EDX spectra of (a) DLA and (b) Fe-DLA; Table S1. The catalytic ozonation process for Reactive Black 5 dye containing wastewater decolorization.
