# Supplementary material for: Catalytic Ozonation of Reactive Black 5 in Aqueous Solution Using Iron-Loaded Dead Leaf Ash for Wastewater Remediation
Source: Molecules. 2024 Feb 13;29(4):836. doi: 10.3390/molecules29040836 (PMC10893045; doi:10.3390/molecules29040836)
Supplement: Supplementary file 1 [file molecules-29-00836-s001.zip › molecules-2785837-supplementary.pdf]

Catalytic Ozonation of Reactive Black 5 in Aqueous Solution Using Iron-Loaded Dead Leaves Ash for Wastewater Remediation

Latif Hussain[1], Farhan Javed[1], Muhammad Wasim Tahir[1], Hafiz Muhammad Shahzad Munir *[2], Amir Ikhlaq[3], Anna Wołowicz*[4]

[1] Department of Chemical Engineering, University of Engineering and Technology, Lahore, Pakistan; latifshifai@gmail.com (L.H.), farhan.javed@uet.edu.pk (F.J.), wasim.tahir@uet.edu.pk (M.W.T)
[2] Department of Chemical Engineering, Khawaja Fareed University of Engineering and Information Technology, Rahim Yar Khan, Pakistan
[3] Institute of Environmental Engineering and Research, University of Engineering and Technology, Lahore, Pakistan; aamirikhlaq@uet.edu.pk (A.I.)
[4] Department of Inorganic Chemistry, Faculty of Chemistry, Institute of Chemical Sciences, Maria Curie Sklodowska University, Maria Curie-Sklodowska Square 2, 20-031 Lublin, Poland
* Correspondence: anna.wolowicz@mail.umcs.pl; Tel.: +48-81-537-57-27 (A.W.); shahzad.munir@kfueit.edu.pk (H.M.S.M.).

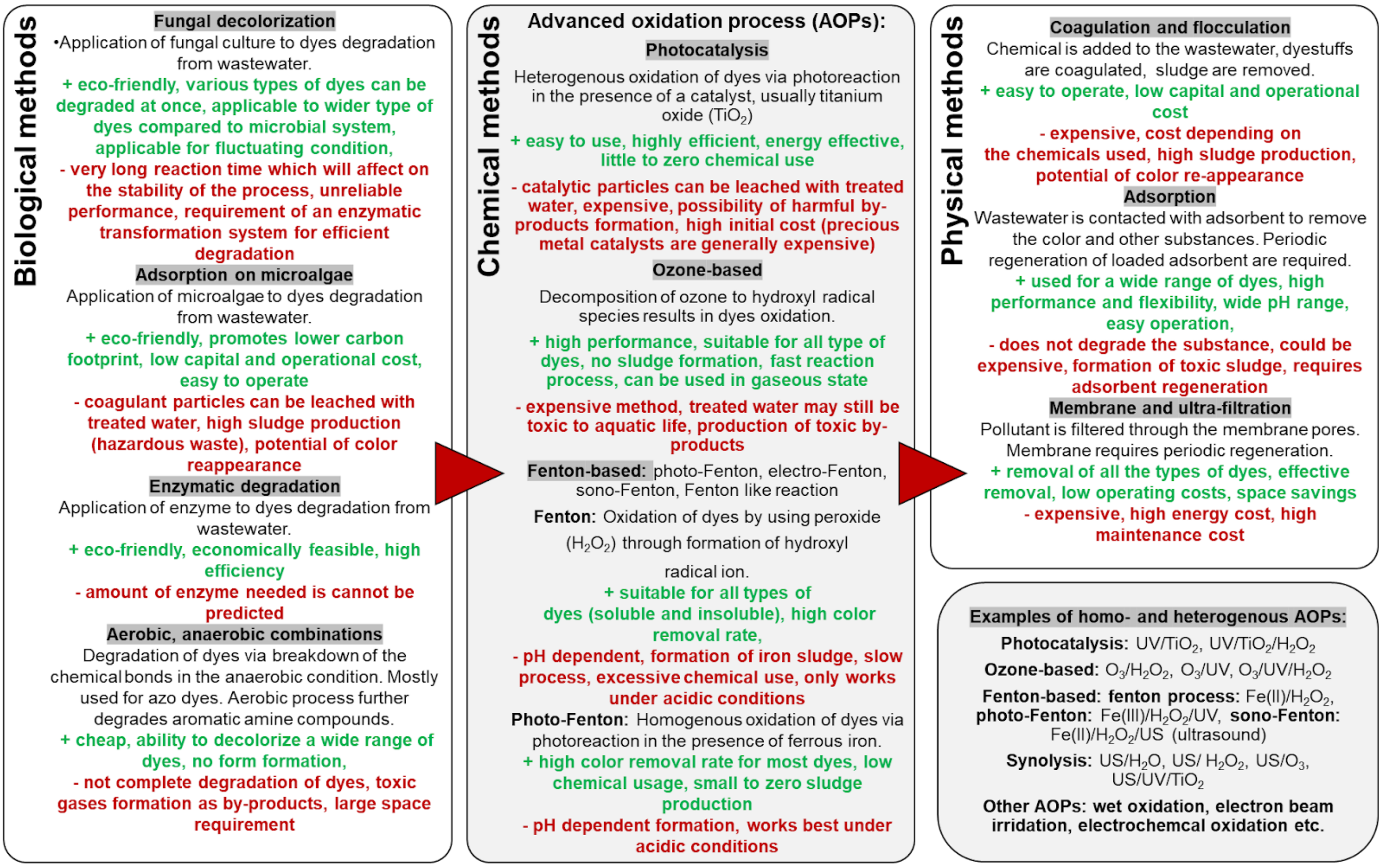

Figure S1. Comparison of advantageous and disadvantageous of dyes containing wastewater decolorization [14-15].

Table S1. The catalytic ozonation process for Reactive Black 5 dye containing wastewater decolorization.

Method	Dye	Results, removal efficiency and other parameters	Ref.
O_3, O_3/UV, O_3/UV/persulfate (PS), and O_3/Ce–Co–O catalyst	Reactive Black 5<br>C_0=100-1000 mg/L	**O_3/Ce–Co–O:** [1]%R_{TOC}= 96% (60 LPH, pH 7, C_0=100 mg/L, catalyst dose=1 g/L, t_r=80 min)<br>**O_3/UV/persulfate:** %R_{TOC}= 90% (60 LPH, pH 12, C_0=100 mg/L, UV intensity=66 W, TOC:PS=1:40, t_r=80 min)	[29]
catalytic ozonation<br>O_3/Ag–Co–O<br>catalyst: silver–cobalt composite oxide	Reactive Black 5<br>C_0=100 mg/L	**O_3 without catalyst**: %R_{TOC}=34%<br>**O_3/Ag–Co–O:** %R_{TOC}=75% (ozone dose=30 mg/min, pH 7, t_r=80 min, catalyst dose=0.5 g/L)	[30]
electro-flocculation, electro-flocculation with catalyst and catalytic ozonation in combination with electro-flocculation<br>catalyst: zeolites modified with $CuMn_2O_4/gC_3N_4$	Reactive Black 5<br>C_0=30-90 mg/L	**electro-flocculation:** 31.18%,<br>**electro-flocculation with catalyst:** 39.27%<br>**catalytic ozonation with electro-flocculation:** 90.31%<br>(ozone dose=1 mg/min, pH 10, C_0=30 mg/L, catalyst dose=1 mg, t_r=30 min, voltage=10 V)	[31]
O_3 assisted electro-coagulation	Reactive Black 5<br>C_0=100-400 mg/L	R_{TOC}=94%, %R_{COD}=60% (C_0=100 mg/L, pH 5.5, current density=10 mA/cm^2, salt concentration=5000 mg/L, T=20°C, ozone dose=20 mL/min, interelectrode distance of 1 cm)	[32]

%R_{TOC} - the total organic carbon (TOC) removal efficiency, %R_{COD} – the chemical oxygen demand (COD) removal efficiency, C_0 – the initial dye concentration, t_r – the reaction time, T – temperature

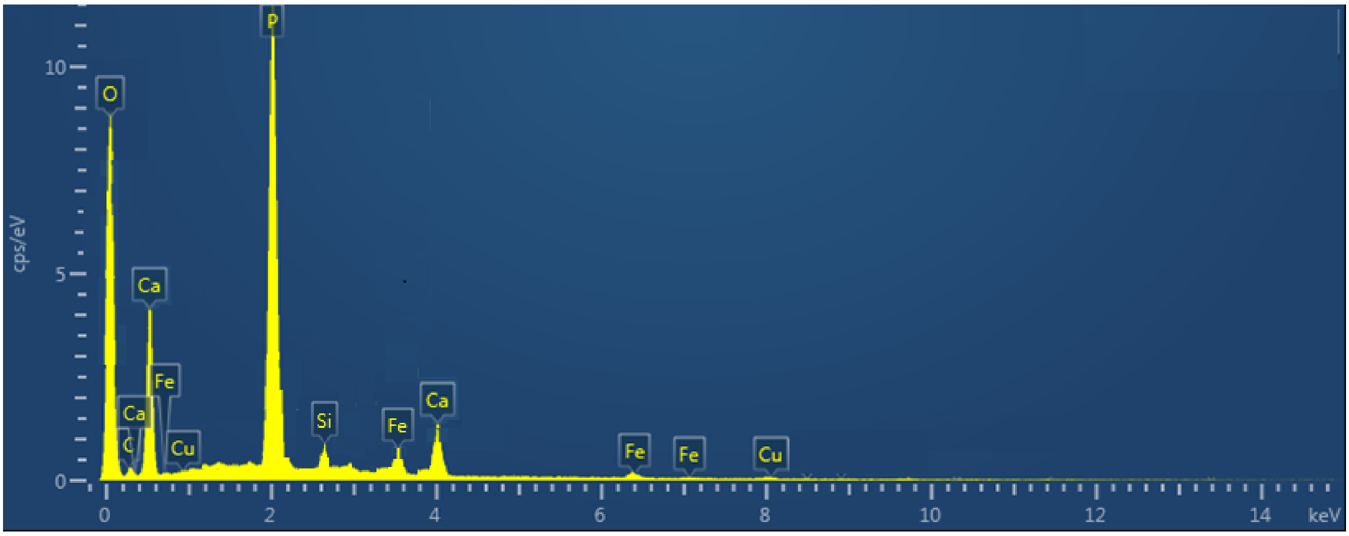

(a)

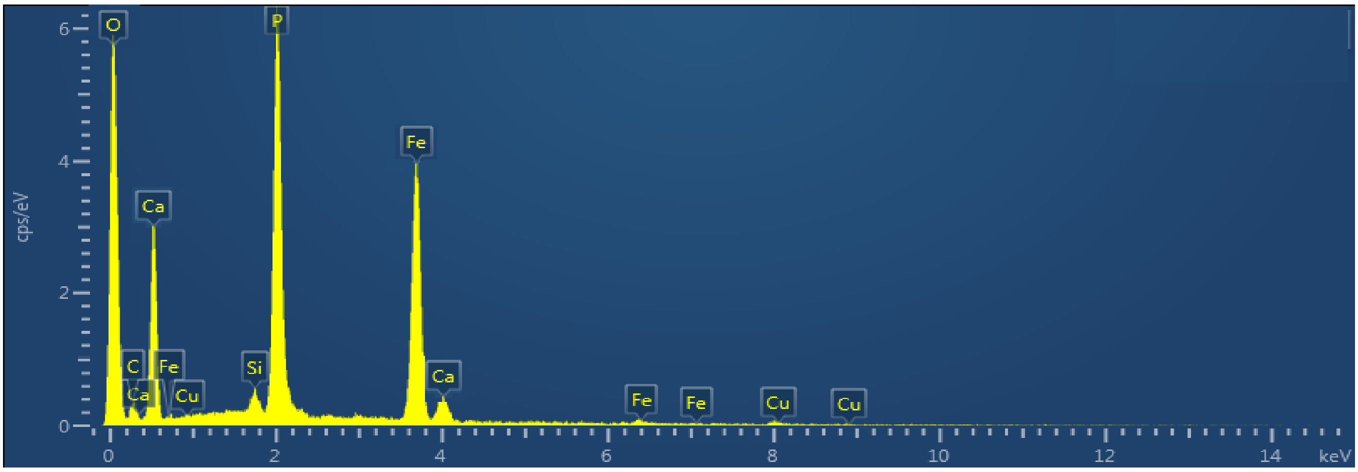

(b)

Figure S2. EDX spectra of (a) DLA and (b) Fe-DLA.
